# The Nordic maintenance care program: case management of chiropractic patients with low back pain – defining the patients suitable for various management strategies

**DOI:** 10.1186/1746-1340-17-7

**Published:** 2009-07-12

**Authors:** Stefan Malmqvist, Charlotte Leboeuf-Yde

**Affiliations:** 1 Department of Health Studies, Faculty of Social Sciences, University of Stavanger, Stavanger, Norway; 2 Norwegian Centre for Movement Disorders, Stavanger University Hospital, Stavanger, Norway; 3 The Research Unit for Clinical Biomechanics, University of Southern Denmark, Odense, Denmark; 4 Institut Franco-Européen de Chiropratique, Paris, France

## Abstract

**Background:**

Maintenance care is a well known concept among chiropractors, although there is little knowledge about its exact definition, its indications and usefulness. As an initial step in a research program on this phenomenon, it was necessary to identify chiropractors' rationale for their use of maintenance care. Previous studies have identified chiropractors' choices of case management strategies in response to different case scenarios. However, the rationale for these management strategies is not known. In other words, when presented with both the case, and different management strategies, there was consensus on how to match these, but if only the management strategies were provided, would chiropractors be able to define the cases to fit these strategies? The objective with this study was to investigate if there is a common pattern in Finnish chiropractors' case management of patients with low back pain (LBP), with special emphasis on long-term treatment.

**Methods:**

Information was obtained in a structured workshop. Fifteen chiropractors, members of the Finnish Chiropractors' Union, and present at the general assembly, participated throughout the entire workshop session. These were divided into five teams each consisting of 3 people. A basic case of a patient with low back pain was presented together with six different management strategies undertaken after one month of treatment. Each team was then asked to describe one (or several) suitable case(s) for each of the six strategies, based on the aspects of 1) symptoms/findings, 2) the low back pain history in the past year, and 3) other observations. After each session the people in the groups were changed. Responses were collected as key words on flip-over boards. These responses were grouped and counted.

**Results:**

There appeared to be consensus among the participants in relation to the rationale for at least four of the management strategies and partial consensus on the rationale for the remaining two. In relation to maintenance care, the patient's past history was important but also the doctor-patient relationship.

**Conclusion:**

These results confirm that there is a pattern among Nordic chiropractors in how they manage patients with LBP. More information is needed to define the "cut-point" for the indication of prolonged care.

## Background

Although lacking in evidence, the term "maintenance care" is well known among chiropractors. Typically, patients who have improved during their initial course of treatment are recommended to extend the treatment period, either in order to prevent further problems (secondary prevention) or to maintain the problem at an acceptable level and to prevent further deterioration (tertiary prevention).

Back problems are recurrent conditions for many. It might well be relevant to choose a long-term management strategy in order to prevent further problems or to keep them under control. However, this is only relevant if the patient gains more than it costs in terms of time and money. Useless or detrimental treatments should obviously not take place, but presently the indications for maintenance care are unclear, as indicated by two literature reviews conducted over the past ten years [1,2]. Furthermore, it is not known if maintenance care has any advantages above the call-when-you-need approach, and if so, if all patients are equally well suited for this approach. Only one randomized clinical trial has been conducted on maintenance care; a pilot study on patients with low back pain (LBP) with non-conclusive results [3]. This lack of evidence has resulted in eager proponents for maintenance care as well as strong adversaries.

Several research groups, co-operating under The Nordic Maintenance Care Program, are presently conducting a number of studies in this area and as an initial step, it became necessary to identify chiropractors' use of maintenance care. Thus, in a previous questionnaire survey, Swedish chiropractors were asked what their strategy would be for nine different cases of LBP, which after a period of treatment had different outcomes. Some had improved, others had varied outcomes including those that had not improved at all. It was possible to choose between six case management strategies that ranged from referring the patient out for a second opinion to maintenance care regardless of the patient's symptoms. It was shown that there was a relatively high consensus on how to manage these nine cases with LBP, particularly when an external opinion (second opinion) was warranted, when the problem was uncomplicated and benign and did not require any further attention, and when the problem was recurrent [4].

The general pattern of management found in the Swedish questionnaire study was confirmed in an additional survey of a group of Danish chiropractors [5]. These were selected to participate in the study because they were known to be proponents of maintenance care and interviewed using the same questionnaire as in the Swedish study. In relation to the use of different case management strategies on patients with LBP, we therefore assumed that there was unspoken understanding amongst chiropractors, regardless of their management approaches, "maintenance care friendly" or relatively unselected practitioners.

However, it was also apparent that there were subgroups of practitioners who had different approaches to the different case scenarios presented in the survey. We were therefore interested in learning more about the rationale for different management strategies, in particular the use of maintenance care. As a consequence, we designed a new study for the group of Finnish chiropractors. Compared to previous studies, instead of providing a number of cases, as we did in the previous two studies, we would present the management strategies. These strategies were the same as those used in the two previous studies. The participants in the new study, who were unaware of the previous two studies, were then asked to describe the patients that would fit these management strategies. The purpose was to investigate if there is a common pattern in Finnish chiropractors' rationale for the use of these case management strategies in patients with LBP, with special emphasis on long-term treatment.

## Methods

Members of the Finnish Chiropractors' Union, present at the annual general assembly and able to participate for the entire session, were invited to participate in this study. The two authors supervised the procedure. Problems with persistent and recurrent LBP were discussed. The participants were then informed that their assistance was needed for further research in this area and that there would be a workshop the following day.

At the workshop, an introduction was given describing the workshop procedure and a basic case was presented, consisting of a hypothetical patient: "A 40-year old man who consults you for low back pain with no additional spinal or musculoskeletal problems and with no other health problems. There are no aggravating factors at work or at home. His X-rays are normal for his age. There are no red flags."

It was then explained that after one month of treatment of this patient, depending on the short-term outcome, the chiropractor would recommend one of six different management strategies. The group was presented with each of these six strategies, one at a time. They were then asked to describe the patient's status and other circumstances at that point in time, which would warrant each of these different choices. The exact type of treatment under consideration was not specified but it was assumed that the participants would use their usual approach, including manipulation, mobilization, advice, exercise and any other adjunctive therapies available in their clinics.

The chiropractors were then divided into five different groups (groups 1 through to 5), with three participants in each. Each group was seated on three chairs and each group had a flip-over board in front of them. For each group, a chairman was selected for the duration of the workshop by. The other participants were divided into two teams, called team 1 and team 2 on the basis of the last digit in their birth date. Each chairman was then provided with two members, one from group 1 and one from group 2. After each management strategy had been discussed, the members of team 1 moved one step in a clockwise fashion and those in team 2 in an anti-clockwise manner. This random mix of participants between the groups was made to avoid dominance of single members. The total number of sessions was six, one for each management strategy.

The main case and the plan for the workshop session were again explained to the group, and the main case was also shown on a screen. The chairman of each group was then provided with a set of notes consisting of six pages; one to be used for each session. Each page had the basic case described at the top, followed by an identical instruction "After one month of treatment, what would this case look like, for you to recommend the following management strategy:" Each page contained one of the following six management strategies:

1. I would refer the patient to another health care practitioner for a second opinion ("Second opinion").

2. I would tell the patient that the treatment is completed but that he is welcome to make a new appointment if the problem returns ("Quick fix").

3. I would not consider the treatment to be fully completed and would try a few more treatments, and perhaps change my treatment strategy, until I am sure that I cannot do anymore ("Try again").

4. I would advise the patient to seek additional treatment whilst following the patient ("External help – keep in touch").

5. I would follow the patient for a while, attempting to prolong the time period between visits until either the patient is asymptomatic or until we have found a suitable time lapse between check-ups to keep the patient symptom-free ("Symptom-guided maintenance care").

6. I would recommend that the patient continues with regular visits regardless of symptoms, as long as clinical findings indicate treatment (e.g. spinal dysfunction/subluxation) ("Clinical findings-guided maintenance care").

In our report, the terms noted in parenthesis after each sentence above were used to describe these strategies, but these brief descriptions were not included in the instruction to the participants.

In order to help rank the participants' responses, they were asked systematically to describe a suitable patient (or several) based on three different aspects: 1. symptoms/findings at the time of the management decision, 2. LBP history in the past year, and 3. other observations.

The groups were given 20 minutes per session to describe a patient that suited the specific management strategy. The chairmen of each group noted the relevant keywords on the board. These keywords could be related to one specific patient, or several different patients. Comments were not noted on the basis of consensus in the group, but could be written down as in a brainstorm session. Each group worked independently. At the end of each session, each group presented their results.

The two supervisors assisted if the groups misunderstood the task at hand or if their comments were difficult to interpret, or if they wrote entire sentences rather than keywords. All groups were assisted for the first case, after which only few extra instructions were needed. A thirty minute coffee break was provided about half way through the procedure.

At the end of the session, the annotated flip-over papers were collected and analyzed by the authors. Each comment was transferred to a separate paper for each of the three aspects (symptoms/findings, LBP history in the past year, and other observation). These replies were then interpreted and identical or very similar keywords added up, and others listed in an attempt to bring similar answers together. The analysis was simple to perform and there were no disagreements between the two researchers. Finally, the numbers of replies for each aspect were counted. On the following day, a summary of the results was provided to the chiropractors, followed by a discussion.

## Results

Fifteen of the 48 members of the Finnish Chiropractors' Union participated in the workshop. They were somewhat hesitant during the first case but lively discussions ensued, and all participants became involved in the process fairly quickly. The results have been reported for each of the three aspects that were answered for each strategy. These three aspects were: 1. symptoms/findings at the time of the management decision, 2. LBP history in the past year, and 3. other observations. In the text, the strategies have been described using the short terms listed in the methods section for each described strategy. The main findings have been summarized in the text based on the background data that are reported in tables.

### Symptoms/findings

1. "Second opinion": There were many different suggestions of why this patient with LBP, after one month, might need to be referred out for a second opinion. Patients who got worse, who developed specific warning signs in relation to neurology or other pathology, and even, patients who had not got better would be considered to be referred out for a second opinion (Table 1).

**Table 1:** A description of patients with LBP who, after 1 month of treatment, fit this management strategy: "I would refer the patient to another health care practitioner for a second opinion".

**General definition of symptoms/findings given as reasons**	**Total number of replies**	**Examples**
Got worse or not better	8	Neurological symptoms Pain

Clinical findings (neurology)	8	Sudden anaesthesia Incontinence Neurological findings Radiating pain Cauda equina Foot drop

Signs of other possible diseases	7	Constitutional signs or symptoms High blood pressure Skin change Night pain/pain at rest Rapid weight loss Unexplained fever

Other aggravating circumstances	4	Antalgia Sciatica Unable to work Referred pain

2. "Quick fix": There were few different suggestions for this case but they all related to absence of symptoms or findings (Table 2).

**Table 2:** A description of patients with LBP who, after 1 month of treatment, fit this management strategy: "I would tell the patient that the treatment is completed but that he is welcome to make a new appointment if the problem returns".

**General definition of symptoms/findings given as reasons**	**Total number of replies**	**Examples**
Absence of symptoms and patient satisfaction	5	No symptoms Patient satisfied

Clinical findings negative	4	Mechanically improved spine Neurological/orthopaedic tests normal Objective findings negative Clinical findings negative

3. "Try again": The explanations of why this patient should be given a second try were mainly centered on failure to improve (sufficiently) or a slight worsening of the situation. However, the clinical situation was not described to be as bad as in case 1 ("Refer out") (Table 3).

**Table 3:** A description of patients with LBP who, after 1 month of treatment, fit this management strategy: "I would not consider the treatment to be fully completed and would try a few more treatments and perhaps change my treatment strategy, until I am sure that I cannot do anymore".

**General definition of symptoms/findings given as reasons**	**Total number of replies**	**Examples**
Not (completely) better or worse	10	Improved but not cured Not better Only a little better New symptoms Slight increase in symptoms Symptoms worse Slightly worse Reoccurrence

Clinical findings	3	Symptom free but clinical findings Recurrent physical findings Antalgia

4. "External help – keep in touch": Respondents seemed to consider sending patients to, mainly, a physiotherapist, a masseur or for physical training, in order to remedy problems with the musculoskeletal system. They also described cases with other health problems and they seemed to be willing to ask for assistance when people either did not improve completely or not sufficiently (Table 4).

**Table 4:** A description of patients with LBP who, after 1 month of treatment, fit this management strategy: "I would advise the patient to seek additional treatment whilst following the case".

**General definition of symptoms/findings given as reasons**	**Total number of replies**	**Examples**
Findings	7	Tight hypertonic muscles Weak unbalanced muscles Body imbalance Instability

Symptoms	6	Better/symptom free New symptoms Less symptoms Insufficient response Stiffness

Other health factors	5	Other new health problem Nutritional deficiency New trauma Sign of inflammation, getting worse with Spinal Manipulative Therapy Local infection, e.g. in foot

5. "Symptom-guided maintenance care": Mainly patients who had improved, subjectively or objectively, were considered for symptom-determined maintenance care (Table 5).

**Table 5:** A description of patients with LBP who, after 1 month of treatment, fit this management strategy: "I would follow the patient for a while, attempting to prolong the time period between visits until either the patient is asymptomatic or until we have found a suitable time lapse between check-ups to keep the patient symptom-free".

**General definition of symptoms/findings given as reasons**	**Total number of replies**	**Examples**
Symptoms	8	Asymptomatic Mild symptoms Good improvement Aggravated by treatment Longer pain free post-treatment periods

Clinical findings	3	Positive clinical findings Objective findings improving and levelling out

6. "Clinical findings-guided maintenance care": The symptoms/findings that seemed to guide this decision were mainly those of incompleteness and a striving for perfection but also signs of recurrent or chronic problems (Table 6).

**Table 6:** A description of patients with LBP who, after 1 month of treatment, fit this management strategy: "I would recommend that the patient continues with regular visits regardless of symptoms, as long as clinical findings indicate treatment (e.g. spinal dysfunction/subluxation)".

**General definition of symptoms/findings given as reasons**	**Total number of replies**	**Examples**
Clinical findings	14	Still subluxated Biomechanical dysfunction still there Better posture Posture changes not yet complete Lumbar lordosis not yet optimal SI stiffness still present Postural imbalance Instability Recurrent severe leg length difference Still antalgic Positive straight leg raise Soft tissue (e.g. trigger points, hypertonicity)

Symptoms	4	Easy onset of LBP Can still get better Recurrent LBP/symptoms Chronic mild neurological signs e.g. stenosis

### LBP history in the past year

All results on this aspect have been reported below.

1. "Second opinion": Three cases of worsening of pain and one of intermittent pain were described, and also one of no previous pain at all in the past year.

2. "Quick fix": The presence of no or very few previous episodes were noted here (n = 4) and also, in one instance, "acute LBP".

3. "Try again": This approach would necessitate that the LBP had been intermittent (n = 4), the past history was also by one group considered to be irrelevant for this approach, but a slow increase in symptoms could also be a possibility

4. "External help – keep in touch": External assistance was an option in all of the groups, if the pain had been intermittent and, for one of the groups, also if the symptoms had increased over the past year.

5. "Symptom-guided maintenance care": All groups would offer this type of maintenance care if the pain was recurrent in the past year and, in one case, also if it had been mild but constant.

6. "Clinical findings-guided maintenance care": Four of the groups would recommend non-symptom guided maintenance care for patients who had recurrent problems, whereas one group did not seem to consider past history to be important for this choice of management strategy (as they had noted "none" as their keyword).

### Other observations

A list of all "other observations" is found in Table 7 and summarised below on the basis of the most frequent replies.

**Table 7:** A description of patients with LBP who, after 1 month of treatment, fit the six management strategies in relation to additional observations.

**Type of strategy**	**Total number of replies**	**Replies**
1. Second opinion	11	Lethargy Malaise Severe weight loss or gain Bad general health Severe stress Untold trauma Illogical pain pattern Sick-leave Psychosocial issues/somatisation

2. Quick fix	9	Bad compliance Lives far away Difficult working hours History of one treatment only Treatment dependent Grateful Looks well now Muscles OK Physically and mentally well balanced

3. Try again	10	Chiropractic treatment successful New clinical findings Recently aggravating factor Post traumatic Minor accident occurred during past month Increased workload Patient somewhat frustrated Bad compliance Patient dissatisfied

4. Exterior help – keep in touch	5	Good compliance Psychosocial problem Easy onset Good response to other therapy Alcohol/drug abuse

5. Symptom-guided maintenance care	8	Compliant patient Satisfied patient Prefers chiropractic to training Increased workload Try to remedy partly inappropriate treatment If the present status is not good enough, time to re-evaluate diagnosis and treatment Compliant

6. Clinical findings-guided maintenance care	11	Satisfied patient Compliant Health-minded patient Athletic Prefers chiropractic to exercises New clinical findings due to change of posture

1. "Second opinion": Some additional clinical findings were described for this patient, all relating to the possibility of other diseases that were unsuitable for chiropractic care.

2. "Quick fix": Most of the comments relating to this strategy explained the inability to continue treatment rather than the reasons for the choice of this management approach. However, there were also some clinical observations included among these reasons.

3. "Try again": The replies for this management strategy were less easily interpreted, spanning from good outcome to the negative aspects of the patient-practitioner relationship.

4. "External help – keep in touch": Again this profile was multifaceted, ranging from good compliance to alcohol/drug abuse. The LBP history in the past year might have been intermittent but there was no clear picture provided for other observations.

5. "Symptom-guided maintenance care": This patient was described as likely to have improved subjectively or objectively, to have had a LBP history of frequent problems and to be satisfied and compliant.

6. "Clinical findings-guided maintenance care": The picture was provided as that of a satisfied, health-oriented and compliant person who prefers chiropractic care to other approaches.

### Overall interpretation of findings – for each management strategy

Based on the findings in the three categories as reported above, we created the following overall profiles:

1. "Second opinion": The patient suitable for referral for a second opinion was described as being likely to have a serious pathology, either neurological or otherwise, to have got worse or, at least, not better over the past month, and his past year LBP history would be one of an intermittent or deteriorating pattern.

2. "Quick fix": The patient whose treatment could be quickly completed was described as having no symptoms and no clinical findings after the first month of treatment, with a past history of no LBP or only few previous episodes. An inability to return for further check-up visits was also mentioned.

3. "Try again": The profile of this patient was less clear, except that an extra attempt or a different approach was considered suitable for patients who had not recovered sufficiently after one month. However, there should be no obvious signs of serious pathology, contrary to strategy 1 ("second opinion").

4. "External help – keep in touch": Again this patient profile lacked a clear definition, although there should be no obvious signs of pathology. A patient with musculoskeletal problems that did not resolve with chiropractic care, or a patient described as not sufficiently improved seemed likely to require a new approach or further attempts. During the workshop, the first impulse seemed to be to think of musculoskeletal based therapies to attempt to remedy the problem (masseur, physical training) but later during the discussion, further possibilities emerged. The LBP history might have been intermittent but there was no clear picture described under other observations.

5. "Symptom-guided maintenance care": Some groups mentioned compliance and patient satisfaction, but the need for extra treatment because of a demanding job was also mentioned, including some replies that seemed to fit better under the "try some more" approach ("try to remedy partly inappropriate treatment" and "not good enough therefore time to re-evaluate diagnosis and treatment").

6. "Clinical findings-guided maintenance care": The chiropractors seemed to strive for "perfection", i.e. trying to improve satisfactory results even further. The LBP history would, as in the case above, be recurrent LBP and the patient being satisfied and compliant.

## Discussion

This study is the third in a series of three, dealing with the same cases and strategies and with special emphasis on maintenance care [4,5]. In the two previous studies, in which the choice of different management strategies was studied in relation to various cases, we noted a fair degree of consensus in how both Swedish and Danish chiropractors matched these two aspects [4,5]. In the present study we attempted to see, if this consensus would work equally well when only a number of management strategies were presented and the chiropractors had to describe the cases that would fit the various management strategies. This attempt appeared to be successful. There seemed to be relative consensus on the rationale for the choice of the various management strategies.

Out of the six case management strategies, this workshop produced a coherent picture of the cases for at least four. Patients likely to be referred out for a second opinion were generally described as having either a non-spinal pathology or a neurological complication that needed to be attended to by another health care practitioner. As a comparison, in the previous Swedish questionnaire survey, "second opinion" was the first choice in two types of patients: those who became gradually worse and another whose status fluctuated for no apparent reason and who also were tired and moody. In other words, these were patients who either did not follow the expected improvement pattern or showed signs of additional problems.

In the present study, the "quick fix" patient was also easily described. The picture, in this case, emerged of a benign case (no or only few previous LBP events) and quick and complete recovery, plus – interestingly – an inability to return for further sessions. In the Swedish study, the "quick fix" option was the first choice in a patient who recovered immediately, with no previous history and no complicating factors. This corresponded well to the patient described in the present study.

We found that the two "maintenance care" strategies were described as suitable for patients who were improved but not "cured", who either needed to be further improved or kept under surveillance. The past history was of importance; it had to be recurrent. The patients' attitudes to treatment were also important, satisfaction and compliance being repeatedly described as necessary.

In relation to prolonged treatment, participants in the Swedish study selected "symptom-guided maintenance care" as first choice in two patients with quick and complete recovery; one who was excessively worried and another with a history of recurrent problems. In yet another survey of Swedish chiropractors, "effectiveness of treatment" and patients "attitude" were considered important inclusion criteria for maintenance care [6].

Less well described, in the present study, were the patients suitable for the "try again" and "external help- keep in touch"- strategies, although the clinical pictures after one month were relatively clearly described as patients who were not sufficiently improved, and the "external help" seemed to be considered for benign musculoskeletal and other health problems. As a comparison, the Swedish data indicated that "try again" was considered in patients who did not improve sufficiently but who did not show any obvious signs of pathology. The "external help – keep in touch" option was never a first choice in the Swedish study.

Studies on maintenance care are sparse and it is important to understand the chiropractors' own opinions on the reasons for this type of treatment strategy. It is also important that chiropractors become aware of the intellectual concepts underlying their clinical decision. The approach that we chose in this study was based on the concept that clinicians should take part in the initial intellectual process of setting up clinical studies in their area of expertise. We also hoped that the method of giving the chiropractors the opportunity to talk about specific clinical issues with different persons would result in an open, unemotional and factual exchange of ideas. During the follow-up session, it was clear that this had succeeded, in that participants became more forthcoming than during previous sessions. It was commented on that the process had been stimulating but also very tiring.

The weaknesses of the study are of course that only a small group of chiropractors took part in the workshop, and that these represented only a small proportion of the Finnish Chiropractors' Union (15/48), and an even smaller group of the Nordic chiropractors. Although these chiropractors were educated at different chiropractic institutions and included both newly graduated and more experienced colleagues, they may not have been representative of the profession. It is also possible that the choice of other case management strategies may have resulted in different responses.

There are several strengths of this study. The workshop design made it possible to accelerate the thought process through structured discussions. Second, the continuous mixing of participants prevented the development of strong partakers who could monopolize the discussion and exert undue influence on the choice of keywords. Third, analysis of the collected information was mainly quantitative to prevent problems of interpretation and there were no issues of disagreement during this process. Finally, this study complemented the two previous surveys of Swedish and Danish chiropractors and the fact that three different populations have now been used to investigate this issue from different angles strengthens our data. The coherent picture that was obtained, based on these three studies, can be interpreted as a validation of the results.

## Conclusion

In conclusion, our findings do confirm that there is a pattern among Nordic chiropractors in how they manage patients with LBP. Our specific interest was to identify the criteria for maintenance care. At this point in time, we can conclude that the patient's past history is important but also other factors that may influence the recommendation of maintenance care, such as the doctor-patient relationship, in particular the patient's attitude to and trust in continued care. However, more information is needed to differentiate the "cut points" for the indications to suggest prolonged care. Also it would be relevant to study further its two main different approaches; the one based mainly on symptoms and the other based mainly (or perhaps exclusively) on the chiropractor's clinical findings.

## Competing interests

The authors declare that they have no competing interests.

## Authors' contributions

Both authors designed and carried out the study. Both undertook the analysis and interpretation of data. The second author wrote the first draft, both authors edited the manuscript, and both authors read and accepted the final version.
